# A pragmatic randomised trial of two counselling models at the Swedish national alcohol helpline

**DOI:** 10.1186/s12888-019-2199-z

**Published:** 2019-07-08

**Authors:** Eleonor Säfsten, Yvonne Forsell, Mats Ramstedt, Kerstin Damström Thakker, Maria Rosaria Galanti

**Affiliations:** 1 Department of Public Health Sciences, Karolinska Institutet, 171 77 Stockholm, Sweden; 2 Centre for Epidemiology and Community Medicine, Stockholm Health Care District, Stockholm County Council, 104 31 Stockholm, Sweden; 3 Department of Clinical Neuroscience, Karolinska Institutet, 171 77 Stockholm, Sweden; 4 The Swedish Council for Information on Alcohol and Other Drugs (CAN), 107 25 Stockholm, Sweden

**Keywords:** Hazardous alcohol use, Harmful alcohol use, Drinking, Telephone helpline, Brief intervention, Counselling, Randomised controlled trial

## Abstract

**Background:**

Alcohol telephone helplines targeting alcohol consumers in the general population can extend the reach of brief interventions while preserving in-person counselling. So far, studies of client outcomes in the setting of alcohol helplines are scarce. This study aims to compare the 6-months alcohol-related outcomes of two counselling models delivered at the Swedish National Alcohol Helpline.

**Methods:**

A pragmatic randomised trial was set up at the Swedish National Alcohol Helpline. First-time callers with current hazardous or harmful alcohol use who contacted the helpline, from May 2015 to December 2017, were invited to participate. Clients were allocated with 1:1 ratio to two groups: (1) brief, structured intervention (*n* = 128), including self-help material and one counsellor-initiated call, and (2) usual care (*n* = 133), i.e. multiple-session counselling using Motivational Interviewing (MI). The primary outcome was a downward change in AUDIT risk-zone between baseline and 6-months follow-up. The analysis followed an intention-to-treat approach.

**Results:**

Recruitment ended in December 2017. At 6-months follow-up, 70% of the enrolled participants had data on the outcome. In the brief, structured intervention (*n* = 107) 68% changed to a lower risk-level, compared to 61% in the usual care group (*n* = 117), yielding a risk ratio (RR) of 1.12 (95% CI 0.93 to 1.37) and risk difference of 0.08 (95% CI -0.05 to 0.20). The total AUDIT score and the scores from the AUDIT consumption questions (AUDIT-C) did not reveal any between-group differences in the mean change at follow-up.

**Conclusions:**

The counselling at the Swedish National Alcohol Helpline was followed by a significant decrease in alcohol use among clients, without clear superiority for either counselling model.

**Trial registration:**

This trial was retrospectively registered with ISRCNT.com (ID: ISRCTN13160878) 18/01/2016.

**Electronic supplementary material:**

The online version of this article (10.1186/s12888-019-2199-z) contains supplementary material, which is available to authorized users.

## Background

The high prevalence of hazardous and harmful alcohol use and the substantial harm attributable to alcohol consumption [1, 2] highlights the need for easily applicable intervention and prevention efforts directed towards at-risk consumers.

In part, the harm caused by alcohol could be prevented by increasing help -seeking in the population with hazardous or harmful alcohol consumption [3]. Only a minority seek formal treatment and help-seeking usually occurs at a relatively late stage [4, 5], partially due to stigma, sceptisism toward treatment alternatives and ignorance of the problems’ severity [6, 7]. Brief interventions (BI) are recognised as effective and cost-effective in primary care populations [8, 9]. However, the extent to which these interventions reach the target population is limited by opportunistic identification [10]; the failure to identify at-risk drinkers [11]; health professional’s attitudes and insufficient implementation strategies [12, 13]. Strategies to reach individuals that would not seek regular treatment includes personalised digital interventions. Recently, a Cochrane review found personalised digital behavioural interventions effective for reducing hazardous or harmful alcohol consumption compared to no or minimal interventions [14]. However, extended counselling does not seem to be more effective for modifying hazardous alcohol use than brief interventions [15]. In the context of telephone helplines the effectiveness of different interventions are unknown.

While telephone-based alcohol counselling via help-lines is gaining momentum, its effectiveness has not been evaluated with experimental studies [16, 17]. The existing studies on brief intervention using telephone counselling typically consist of clinical populations, i.e. not seeking help for alcohol problems per se. These studies suggest that the telephone may be an effective mode for alcohol counselling [18] [19] [20] [21], a suggestion supported by previous reviews on telephone counselling in mental health and addiction [22, 23]. Further, the scant evaluation of alcohol helplines limits the knowledge about effective models of delivery.

Population-based telephone counselling, combines ease of access and anonymity with the advantages of in-person individual counselling, thus reducing potential barriers to treatment seeking. Last but not least, telephone helplines may be cost-effective for the delivery of in-person counselling.

The Swedish National Alcohol Helpline (SAH), operating since 2007, offers a unique possibility to study telephone counselling aimed at the general population. From the very start, a strong emphasis was put on developing and providing the most cost-effective counselling setting. Previous observational studies at SAH offered a suitable “proof of concept” of the usefulness of the service [24, 25]. This study seeks to move the agenda further, comparing the effects of two counselling models on hazardous and harmful alcohol use within the SAH. 1) A brief structured intervention consisting of self-help material combined with one counsellor-initiated call, and 2) usual care, i.e. multiple-sessions of Motivational Interviewing (MI) with components of cognitive behavioral therapy (CBT). The alternative hypothesis was that the brief structured intervention would be more effective than usual care in promoting change in a client’s alcohol drinking habits.

## Methods

A protocol of the trial, including full information of the design and methods, has been published and is summarised here [26].

### Study design

A pragmatic randomised controlled trial was initiated in 2015 at the SAH. The Ethical Review Board of Stockholm, Sweden approved the study (DNR 2014/1732–31/5), and the corresponding protocol was registered in the ISRCTN registry (ID: ISRCTN13160878). The analytical approach to hypothesis testing has been changed [26] from that reported in the trial register from non-inferiority to superiority due to a lower recruitment rate then what was expected at inception.

### Recruitment of participants and random allocation

Participants were clients from the general population seeking help at the SAH for at least hazardous alcohol use. Counsellors assessed the eligibility of clients at their first contact, before informing about the study. Eligible clients who expressed interest in participating were contacted by telephone within a week by trained interviewers not involved in the counselling at the helpline. The interviewers performed the following sequence of tasks: 1) obtained formal consent, 2) conducted the baseline interview, and 3) opened a sequentially numbered, sealed envelope containing the results of the randomization algorithm and communicated the group allocation to the participant. Clients were allocated with 1:1 ratio to the two groups. The research coordinator prepared the envelopes containing the computer-generated allocation sequence. Detailed information on the randomisation and recruitment process can be found in the study protocol [26]. The enrolment period was from May 27, 2015, to December 15, 2017, while the 6 months follow-up was completed between December 5, 2015 and June 20, 2018.

First-time callers or callers with a washout period of at least 1 year since last SAH contact, that were adults (≥18 years) who spoke Swedish and sought help for at least hazardous alcohol use were eligible to participate in the trial. Hazardous alcohol use was identified by the Alcohol Use Disorders Identification Test (AUDIT), using a cut-off of 6 and 8 points or more for women and men respectively [27]. Clients were excluded if the counsellor made the assessment that a caller required referral to treatment for severe alcohol problems or if the client reported the concurrent use of illicit drugs, or the suffering from severe psychiatric conditions or other acute health problems that required medical attention. Further, counsellors refrained from informing clients about the study if the overall assessment at the first call indicated that clients were not able at that time to understand the conditions for participation in the study.

### Trial groups

#### Brief structured intervention

The brief structured intervention includes a self-help booklet and one counsellor-initiated call (i.e. proactive). The self-help booklet is based on CBT and provides a step-by-step guide to change alcohol use. In brief, it aims to increase motivation to change, initiate reflection, facilitate goal-setting and self-monitoring, and provide suggestions on how to build resistance skills. The self-help booklet was delivered by e-mail or ordinary mail or could be downloaded from a password-protected website. Two weeks after dispatching the self-help booklet a counsellor contacted the participant. The counselling in the proactive call was based on a brief manual with the focus of facilitating the use of the material. Counsellors were asked to document the delivery of the proactive call as 1) completed according to the manual, 2) completed with other content, i.e. usual MI counselling with other focus than the material, or 3) not completed, i.e. not rescheduled within 2 weeks from the first contact or could not be reached after five attempts.

After the proactive call, no further contacts were initiated by the counsellor. However, clients were not prohibited from calling the SAH again if they felt the need to do so. If additional contacts were initiated by the client, usual counselling was provided.

#### Usual care

The usual care at the SAH builds on MI with components of CBT. The purpose is to promote clients motivation to change, develop resistance skills and prevent relapses. The sessions vary in number, duration and mode, (i.e. reactive or proactive), and is determined by the counselling need of each client. The counselling is tailored according to the stage of change of the client (contemplation, preparation, action and maintenance). In case of a fifth session the counsellor and the client make a joint evaluation of whether the client has reached his or her alcohol-related goals or if there is a need for further support. When necessary either a maximum of two additional reactive calls may be offered or the client is referred to a specialised treatment provider. After each session the core content and the client’s alcohol-related goals are registered in an electronic record, to enable consistent counselling between sessions.

### Data collection

Data was collected by structured interviews at baseline and at 6 months follow-up. At baseline, the interview covered questions of demographics (sex, age, education, employment, and living arrangements), and social support. Additional information included co-morbidity, indicators of general health, and help-seeking for alcohol-related problems (past 6 months). To screen for major depressive episodes (MDE, past 2 weeks) and generalized anxiety disorder (GAD, past 6 months), the measured mental-health problems, two sections of the M.I.N.I. (Mini-International Neuropsychiatric Interview M.I.N.I.) were used [28, 29]. Indicators of general health status were: sick-leave during the past 6 months, and self-assessed health, the latter measured by “How would you rate your overall health status?” in which responses were collapsed into three alternatives: ‘Very good to Good’, ‘Fair’, ‘Bad to Very bad’) [30]. Motivation to change was measured at baseline as it could be a predictor of the outcome [31, 32]. This was measured by the Readiness to change ruler ranging from 0 to 10, where 10 defines very high degree of readiness [31]. At baseline, AUDIT was primarily completed at the first call, since this scale is usually administered at the SAH as an essential tool for the diagnosis of the severity of alcohol problems. In some cases when AUDIT was not completed at the first call, it was administered in the subsequent baseline interview. Data retrieved from the client record at the helpline included AUDIT score at baseline and number and length of calls. A call was considered a counselling session if it lasted at least 5 min. AUDIT was re-administrated at the 6-month follow-up interview. Interviewers who performed the follow-up interviews were blinded as to the participant’s experimental group.

### Outcome definition and measures

Problematic alcohol use was measured by the Alcohol Use Disorders Identification Test (AUDIT) [27]. AUDIT is a validated instrument, sensitive to problematic alcohol use in the lower end of the spectrum [33, 34]. Risk levels were based on cut-offs described in the Swedish AUDIT manual: ‘low-risk use’ (score 0–5 women; 0–7 men), ‘hazardous use’ (score 6–13 women; 8–15 men), ‘harmful use’ (score 14–17 women; 16–19 men), and ‘probable dependence’ (score ≥ 18 women; ≥20 men) [27]. Further, the AUDIT-C was used to assess frequency and quantity of drinking. This measure includes the first three questions of the AUDIT instrument and has a maximum score of 12.

The primary outcome was defined as any downward shift in AUDIT risk level at 6 month follow-up, i.e. a ‘downward change in risk level compared to baseline’. An upward shift or no change in risk level was defined as ‘no downward change in risk level’. Secondary outcome measures were: 1) change to low risk level according to the AUDIT cut-offs, 2) mean change in the total AUDIT score, and 3) mean change in AUDIT-C at 6-month follow-up, this latter representing a measure of current alcohol use.

### Statistical analysis

The analysis was carried on as a modified intention-to-treat analyses (ITT), i.e. participants with outcome information at 6-month follow-up were analysed according to their randomly allocated counselling model, irrespective of the counselling received. To assess the impact of attrition a sensitivity analysis was performed assuming that AUDIT risk level among those lost to follow-up did not change or was worsened as compared to their baseline score; and by the last observation carried forward (LOCF) scenario, i.e. assuming that participants lost to follow-up did not change their baseline AUDIT score.

A descriptive analysis of the implementation of the two counselling models was also conducted. Baseline characteristics of participants in the two trial groups are presented as percentages for categorical variables and as means and standard deviations (SD) for continuous variables. The distribution of selected characteristics at baseline in the two trial groups was reviewed in order to assess the success of the random assignment. We used general linear models (GLM) as we did not adjust for any covariates, assuming differences in baseline characteristics to arise by chance. The treatment effect was estimated as risk ratio (RR) using the probability of downward change in AUDIT risk level as the outcome (pre-specified); as well as risk difference (RD) and 95% confidence interval (CI). Additionally, we estimated the risk ratio (RR) and 95% confidence interval (CI) using the probability of change to low risk level at follow-up as the outcome. Further, we analysed the between-groups difference in the mean change in AUDIT and AUDIT-C score from baseline to follow-up using a t-test. The level of conventional statistical significance was set to *p* = 0.05. All analyses were performed using Stata 14.1.

## Results

**Fig. 1:**
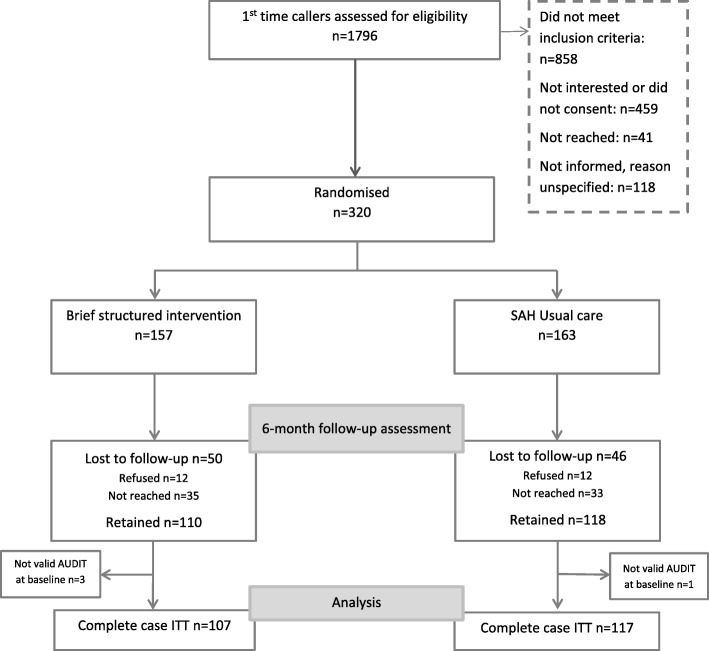
Flowchart over the recruitment, randomisation and follow-up

In total 1796 first time callers were screened for eligibility during the recruitment period (see Fig. 1). Of these, 816 met the eligibility criteria and were informed about the study, and 320 (39% of the eligible) agreed to participate and were randomised. Out of the randomised participants, 224 participants could be followed-up for outcome information at 6-month. In the brief structured intervention 68% were retained while 72% were retained in usual care. Participants with missing AUDIT score (*n* = 2) or low-risk use (*n* = 2) at baseline were excluded from further analysis.

Table 1 shows the baseline characteristics of retained participants by trial group, as compared to those not followed. The not followed participants tended to be younger and employed. Additionally, the proportion defined as ‘probably dependent’ was higher among participants lost to follow-up (66%) than among those who completed the 6-month follow-up (49%), Table 2. Table 2 shows that the trial groups were balanced at baseline based on demographic characteristics with the only exception being self-assessed general health, i.e. good to excellent health was more frequently reported in the brief structured intervention (71% vs 59%). The readiness to change ruler (range 1–10), similarly indicated high motivation in the two groups mean (sd); brief structured intervention 9.3 (1.6) vs usual care 9.3 (1.2). About one-third of the sample displayed MDE or GAD at baseline (brief structured intervention 27% vs usual care 33%). AUDIT risk levels at baseline were similar between the two groups. In the analytical sample it was defined as hazardous for 18% vs 21%, as harmful for 33% vs 31% and as probable dependence for 50% vs 48% of the participants in the brief structured intervention and usual care respectively. Additional file 1 display sociodemographic and health related characteristics by trial group for the complete sample.

**Table 1:** Sociodemographic characteristics at baseline of the followed participants by trial group, and the not followed participants

	Brief structured intervention *n* = 107	Usual care *n* = 117	Total sample *n* = 224	Not followed *n* = 96
Age (mean, sd)^a^	48.5 ± 13.9	49.7 ± 13.8	49.1 ± 13.8	45.6 ± 13.0
Sex, n (%)				
Women	31 (29.0)	29 (24.8)	60 (26.8)	36 (37.5)
Men	76 (71.0)	88 (75.2)	164 (73.2)	60 (62.5)
Employment status, n (%)^a^				
Unemployed	26 (24.5)	37 (32.2)	63 (28.5)	13 (13.7)
Employed	80 (75.5)	78 (67.8)	158 (71.5)	82 (86.3)
Education, n (%)^a^				
Primary	12 (11.4)	9 (7.8)	21 (9.6)	9 (9.4)
Secondary	33 (31.4)	50 (43.5)	83 (37.7)	45 (46.9)
Post-secondary	60 (57.1)	56 (48.7)	116 (52.7)	42 (43.8)
Living arrangement, n (%)				
Living alone (yes)	26 (24.3)	27 (23.1)	53 (23.7)	18 (18.8)
Cohabiting with partner (yes)	70 (65.4)	76 (65.0)	146 (65.2)	70 (72.9)
Living with children (yes)	42 (39.3)	33 (28.2)	75 (33.5)	39 (40.6)
Social support during crisis, n (%)^a^				
Always	3 (2.8)	7 (6.0)	10 (4.5)	9 (9.4)
Occasionally	23 (21.7)	33 (28.4)	56 (25.2)	23 (24.0)
Never	80 (75.5)	76 (65.5)	156 (70.3)	64 (66.7)

^a^Numbers may not sum up to the total because of a few missing values

**Table 2:** Baseline AUDIT score and health-related characteristics of the followed (analytical sample) participants by trial group and not followed participants

	Brief structured intervention *n* = 107	Usual care *n* = 117	Total sample *n* = 224	Not followed *n* = 96
AUDIT score^a^ mean (sd)	19.6 ± 5.2	19.1 ± 5.8	19.4 ± 5.5	20.9 ± 6.0^d^
AUDIT risk level, n (%)^a, b, d^				
Low risk	0	0	0	2 (2.1)
Hazardous	19 (17.8)	25 (21.4)	44 (19.6)	13 (13.8)
Harmful	35 (32.7)	36 (30.8)	71 (31.7)	17 (18.1)
Probable dependence	53 (49.5)	56 (47.9)	109 (48.7)	62 (66.0)
Readiness ruler (1–10) mean (sd)	9.3 ± 1.6	9.3 ± 1.2	9.3 ± 1.4	9.6 ± 1.1
MDE & GAD, n (%)^c^	28 (26.7)	38 (33.0)	66 (30.0)	35 (37.6)
Self-assessed health, n (%)				
Very poor to poor	3 (2.8)	4 (3.4)	7 (3.1)	7 (7.3)
Fair	28 (26.2)	44 (37.6)	72 (32.1)	25 (26.0)
Good to excellent	76 (71.0)	69 (59.0)	145 (64.7)	64 (66.7)
Past 6-month sick-leave, n (%)^a^				
0–7 days	68 (80.0)	77 (88.5)	145 (84.3)	67 (78.8)
≥ 8 days	17 (20.0)	10 (11.5)	27 (15.7)	18 (21.2)
Past 6-month help-seeking for alcohol problems, n (%)				
Health care^a^ (yes)	12 (11.3)	17 (14.5)	29 (13.0)	19 (19.8)
Other care (yes)	7 (6.5)	10 (8.5)	17 (7.6)	14 (14.6)
Medication for alcohol dependence (yes)	5 (4.7)	9 (7.7)	14 (6.3)	9 (9.4)

^a^Number may not sum up to the total because of a few missing values

^b^AUDIT score (women; men) 1 (0–5; 0–7), 2 (6–13; 8–15) 3 (14–17; 16–19) 4 (18–40;20–40)

^c^Major depressive episode and generalized anxiety disorder

^d^*n* = 94, including baseline AUDIT scores below the threshold for hazardous use (*n* = 2)

In the brief structured intervention, 73% of the participants in the analytical sample received the proactive call according to the protocol and 5% received a modified proactive call. Among the retained participants, the mean number (sd) of contacts recorded at the SAH was 1.8 (0.8) in the brief structured intervention, and 3.4 (2.4) in usual care. The time spent in counselling was on average 43 (26) minutes in the brief structured intervention and 89 (76) minutes in the usual care.

In the group receiving the brief structured intervention 68% displayed a downward shift in AUDIT risk level at follow-up compared to 61% in the group receiving the usual care, (Table 3). Consequently, the probability of downward change was 12% higher in the brief structured group than in the usual care group but the confidence intervals included the null (RR 1.12, 95% CI 0.93 to 1.37). The mean decrease in AUDIT score was 7.9 in the brief structured intervention and 7.1 in the usual care group, with a between-group difference of 0.8 points (95% CI − 1.0 to 2.8) (Table 3). Regarding alcohol consumption, the mean change in AUDIT C displayed no between-group differences 0.2 (− 0.5 to 0.9) (AUDIT C), (Table 3). The proportion who changed to low-risk was 30% in the brief structured intervention and 26% in the usual care (RR 1.17, 95% CI 0.76 to 1.78), (data not shown in the table). In the total sample 8% (*n* = 19) were abstainers at follow-up, and 6% (*n* = 13) had changed to a higher risk level, with no difference between the groups.

**Table 3:** Risk ratio and risk difference of transition to lower AUDIT category and change in AUDIT mean scores from baseline to 6 month follow-up, Intention to treat analysis (ITT)

		AUDIT risk level at follow-up %				Change in AUDIT risk level		
	n	l	II	III	IV	%	Risk ratio (95% CI)	Risk difference (95% CI)
Brief structured intervention	107	29.9	40.2	13.1	16.8	68.2	1.12 (0.93 to 1.37)	0.08 (−0.05 to 0.20)
Usual care	117	25.6	40.2	14.5	19.7	60.7	Reference	
	n	Baseline mean (sd)		Follow-up mean (sd)			Mean difference (95%CI)	Mean difference between groups (95% CI)
AUDIT score								
Brief, structured intervention	107	19.7 (5.8)		11.7 (6.9)			−7.9 (−9.3 to −6.4)	0.87 (−1.0 to 2.8)
Usual care	117	19.1 (5.7)		12.1 (7.3)			−7.1 (−8.4 to −5.7)	
AUDIT C score								
Brief, structured intervention	107	7.8 (2.2)		5.0 (2.8)			−2.8 (−3.3 to −2.3)	0.20 (−0.53 to 0.92)
Usual care	117	7.6 (2.0)		5.0 (2.8)			−2.6 (−3.1 to −2.1)	

AUDIT risk levels: I ‘low risk’ II ‘hazardous III ‘harmful IV ‘probable dependence’

Since the attrition was similar in the two counselling groups the risk ratio in the alternative scenario (i.e. lost to follow-up did not change or was worsened as compared to their baseline score) was close to that obtained in the available case ITT analysis (RR 1.08; 95% CI 0.85 to 1.38). In the LOCF scenario, the mean difference between groups remained in the same direction as in the ITT analysis in-between group difference 0.4 points (− 1.14 to 1.95).

**Fig. 2:**
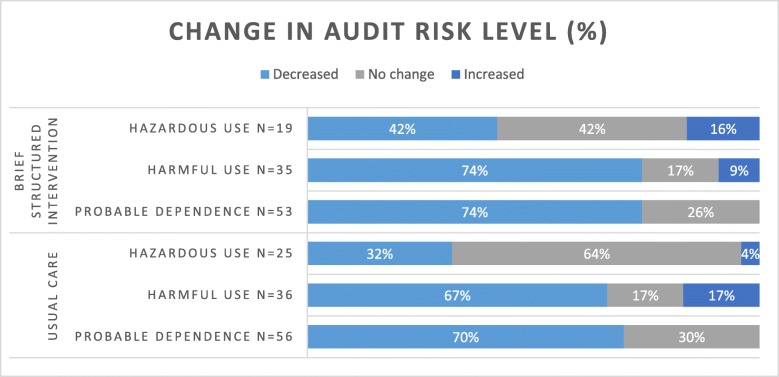
Change in AUDIT risk level (%) between baseline and 6 month follow-up, by intervention group

Figure 2 shows the proportion of downward, upward and no change in AUDIT risk zones by baseline risk level and intervention group. Hazardous users were more likely to shift to low-risk use in the brief structured intervention than in the usual care group, while the proportion downward shift in harmful and probable dependent risk levels was similar between treatment groups.

## Discussion

In this pragmatic trial of counselling at the SAH, there was no evidence of superiority of either intervention (brief structured intervention or usual care) towards the other. However, the results of the downward shift in AUDIT risk level indicated a trend in favour of the less labour intensive structured intervention. Both groups in this trial reduced their AUDIT risk levels indicating reductions in hazardous and harmful alcohol use over time. Overall, about 2/3 of the participants changed to a lower AUDIT risk level, and about 1/3 changed to low-risk in the two counselling groups at 6 months follow-up.

Population-based telephone helplines are nowadays widely implemented for the counselling of problematic alcohol use. However, scientific evaluations of their potential to change alcohol consumption are rare [23], and have mainly described the services [35], or been observational [24, 35]. While the effectiveness of telephone counselling in smoking cessation is well-documented [36], we found only one comparable trial aiming to evaluate a population-based telephone counselling for problematic alcohol use [16]. In contrast to our findings, Signor et al. found that brief MI counselling was more effective than the control condition (self-help booklet combined with brief advice) for abstinence at 6 months follow-up (70% vs 41%). However, this study suffered from high attrition (77%); also, the participants had severe alcohol problems at inception and often reported the concurrent use of illicit drugs [16].

Our study indicated that a brief structured intervention including a self-help material and one proactive call might be as beneficial as the more counselling intensive counterpart. Bibliotherapy is a recognised method to reduce alcohol consumption [37, 38], especially when coupled with feedback sessions [39, 40]. However, a recent study found no additional effects by adding MI telephone sessions to bibliotherapy alone among self-referred problematic alcohol users, without severe dependence [41]. In this study, interventions led to similar reductions in alcohol use, with constant improvements seen between post-treatment and 12 months. However, the sample size was small (*n* = 111), thus differences between groups may have not been detected [41]. With the exception of these studies, telephone counselling for problematic alcohol use has primarily been studied in RCTs of clinical populations identified by screening interventions in healthcare settings including participants with lower problem severity than among clients contacting the SAH [18, 19].

There are several potential explanations of the null finding in this study. First, the effect of the two counselling models might truly be comparable. In fact, many clients calling the SAH are likely to be highly motivated to change their behaviour, and probably already started the process of change before the first contact. Highly motivated individuals may benefit from a structured intervention that offers planning and support to implement personal strategies to reach their goals. Second, at the initial contact, many participants were screened for alcohol use and received assessment feedback from a counsellor, which might have enhanced motivation and thus constitute an active intervention component per se. Thus, the effect might be due to assessment reactivity rather than the added intervention components [42, 43]. Third, the low sample size may have entailed low power to detect small differences between groups as statistically significant.

Despite that the intended target population of the SAH consists of problematic alcohol users at the lower end of the spectrum [26], a high proportion of participants was classified as probably dependent at baseline, reflecting the average level of problematic alcohol consumption in SAH clients overall. Similar to previous studies, the attrition was related to the severity of problematic drinking [44]. This suggests that clients with ‘probable dependence’ would benefit from other support strategies than those offered at the helpline.

### Strengths and limitations

The retention in this study was reasonably high compared to previous studies in the field [16, 45], with no differential loss to follow-up between the two counselling groups. The similar retention between experimental groups, besides reassuring about selection bias also suggests that both counselling models were acceptable to many of the SAH clients. The study protocol was implemented as planned, and the delivery of the brief, structured intervention was ascertained by regular meetings between the employees at SAH and the study team. Further, the personnel conducting the interviews were not part of the SAH and blinded to the client’s group assignment. The random allocation was concealed.

Limitations of this study include the low recruitment rate, as only 46% of first-time callers were assessed as eligible. This was due to a “service-based” screening, which left great freedom of decision to each counsellor at the SAH. In fact, counsellors assessed the eligibility of each caller not only based on the pre-defined criteria, but also taking into account the complexity of the problem presented by the individual at the moment of the call. For instance, clients who were too emotionally disturbed at the time of the call, or who reported serious social consequences of their drinking calling for immediate actions, such as involvement of minors were judged unreceptive to questions about the study, therefore discarded as not eligible. Of the eligible and only 39% participated. Besides resulting in a small sample size, this selection certainly limits the generalizability of the results. However, age and alcohol use profile of the participants in this study were comparable to those of the overall SAH clients [26].

Some contamination of the experimental groups may have occurred since the counsellors interacting with the clients were the same in both groups. However, the average number and length of the sessions were substantially higher in the usual care group, confirming that the brief, structured intervention was less labour intensive, as expected. Additionally, the documentation of the content in the proactive call did not indicate major deviations from the study protocol. As in most trials of behavioural modification the endpoints in this study rely on self-reported data, thus are potentially prone to information bias. However, the follow-up assessment was conducted by interviewers not involved in the counselling, which should at least limit the risk of socially desirable reports.

The main outcome measure AUDIT has traditionally been scored as a three-factor screening instrument, however a review of studies support a two factor model; patterns of intake/consumption (item 1–3) and alcohol problems (item 4–9) [46]. Therefore a change in the AUDIT score may not necessarily depend on changes in alcohol consumption, which is instead captured by the AUDIT-C subscale. This was included as an additional sensitivity analysis, yielding results in line with those obtained from the complete AUDIT.

As this was a trial enrolling callers who sought help for alcohol use at a well-established service, a no-treatment control condition was considered unethical. Therefore, the conclusions of this study apply to the model of the counselling, and not to the effectiveness of the helpline in comparison with other counselling settings or a no-treatment control group.

## Conclusions

A brief structured intervention did achieve favourable changes in problematic alcohol use among clients of the Swedish National Alcohol Helpline that were similar to those of a more labour intensive MI-based telephone counselling. Both approaches were followed by significant changes in clients’ AUDIT risk levels, comparable to the effect of other interventions. The results suggest that both approaches are feasible and acceptable for clients seeking help at the helpline.

## Additional file

Additional file 1: **Table S1.** Baseline characteristics by trial group. **Table S2.** Baseline AUDIT score and health-related characteristics by trial group. Sociodemographic characteristics Baseline AUDIT score and health-related characteristics of the participants at baseline by trial group, total sample. (DOCX 17 kb)

## Data Availability

The datasets generated and/or analysed during the current study are not publicly available due to ethical reasons but are available from the corresponding author on reasonable request.

## Abbreviations

AUDIT: Alcohol Use Disorders Identification Test
CBT: Cognitive behavioral therapy
CI: Confidence interval
GAD: Generalized anxiety disorder
M.I.N.I.: Mini-international neuropsychiatric interview
MDE: Major depressive episodes
MI: Motivational Interviewing
RD: Risk difference
RR: Risk ratio
SAH: Swedish National Alcohol Helpline
Sd: Standard deviation
