# Supplementary material for: A pragmatic randomised trial of two counselling models at the Swedish national alcohol helpline
Source: BMC Psychiatry. 2019 Jul 8;19:213. doi: 10.1186/s12888-019-2199-z (PMC6615184; doi:10.1186/s12888-019-2199-z)
Supplement: Supplementary file 1 — Table S1. Baseline characteristics by trial group. Table S2. Baseline AUDIT score and health-related characteristics by trial group. Sociodemographic characteristics Baseline AUDIT score and health-related characteristics of the participants at baseline by trial group, total sample. (DOCX 17 kb) [file 12888_2019_2199_MOESM1_ESM.docx]

Table S1. Baseline characteristics by trial group

|  | Brief structured intervention  n=157 | Usual care n=163 | Total sample n=320 |
| --- | --- | --- | --- |
| Age (mean, sd)^a^ | 47.6±13.8 | 48.4±13.5 | 48.0±13.7 |
| Sex, n (%) |  |  |  |
| Male | 107 (68.2) | 117 (71.8) | 224 (70.0) |
| Female | 50 (31.9) | 46 (28.2) | 96 (30.0) |
| Employment status, n (%) ^a^ |  |  |  |
| Unemployed | 33 (21.3) | 43 (26.7) | 76 (24.1) |
| Employed | 122 (78.7)) | 118 (73.3)) | 240 (75.9) |
| Education, n (%)^a^ |  |  |  |
| Primary | 16 (10.3) | 14 (8.7) | 30 (9.5) |
| Secondary | 55 (35.5) | 73 (45.3) | 128 (40.5) |
| Post-secondary | 84 (54.2) | 74 (46.0) | 158 (50.0) |
| Living arrangement, n (%) |  |  |  |
| Living alone (yes) | 38 (24.2) | 33 (20.3) | 71 (22.2) |
| Cohabiting with partner (yes) | 104 (66.3) | 112 (68.7) | 216 (67.5) |
| Living with children (yes) | 60 (38.2) | 54 (33.1) | 114 (35.6) |
| Social support during crisis, n (%)^a^ |  |  |  |
| Always | 10 (6.4) | 9 (5.6) | 19 (6.0) |
| Occasionally | 34 (21.8) | 45 (27.8) | 79 (24.8) |
| Never | 112 (71.8) | 108 (66.7) | 220 (69.2) |

Table S2. Baseline AUDIT score and health-related characteristics by trial group

|  | Brief structured intervention  n=157 | Usual care n=163 | Total sample  n=320 |
| --- | --- | --- | --- |
| AUDIT score ^a^ mean (sd) | 20.1 ± 5.7 | 19.6 ± 5.7 | 19.8 ± 5.7 |
| AUDIT risk level, n (%) ^a, b^ |  |  |  |
| Low risk | 2 (1.3) | 0 | 2 (0.6) |
| Hazardous | 27 (17.3) | 30 (18.5) | 57 (17.9) |
| Harmful | 41 (26.3) | 47 (29.0) | 88 (27.7) |
| Probable dependence | 86 (55.1) | 85 (52.5) | 171 (53.8) |
| Readiness ruler (1-10) mean (sd) | 9.4 ± 1.5 | 943 ± 1.1 | 9.4 ± 1.3 |
| MDE & GAD, n (%) ^c^ | 48 (31.2) | 53 (33.3) | 101 (32.3) |
| Self-assessed health, n (%) |  |  |  |
| Very poor to poor | 7 (4.5) | 7 (4.3) | 14 (4.4) |
| Fair | 39 (24.8) | 58 (35.6) | 79 (30.3) |
| Good to excellent | 111 (70.7) | 98 (60.1) | 209 (65.3) |
| Past 6-month sick-leave,  n (%)^a^ |  |  |  |
| 0-7 days | 106 (82.8) | 106 (82.2) | 212 (82.5) |
| ≥ 8 days | 22 (17.2) | 23 (17.8) | 45 (17.5) |
| Past 6-month help-seeking for alcohol problems, n (%) |  |  |  |
| Health care ^a^  (yes) | 21 (13.5) | 27 (16.6) | 48 (15.1) |
| Other care (yes) | 16 (10.2) | 15 (9.2) | 31 (9.7) |
| Medication for dependence (yes) | 10 (6.4) | 13 (8.0) | 23 (7.2) |
